# Addressing Filicide in Ghana: Linking Cultural Understanding With the Law Against Filicide. Does the Law Work?

**DOI:** 10.3389/fpsyg.2022.928963

**Published:** 2022-07-15

**Authors:** Alhassan Abdullah, Margarita Frederico, Felix Mensah, Hajara Bentum, Yihang Wang, Jennifer Litela Asare

**Affiliations:** ^1^Department of Social Work and Social Administration, The University of Hong Kong, Hong Kong, Hong Kong SAR, China; ^2^Department of Sociology, Haverford College, Haverford, PA, United States; ^3^Department of Occupational Therapy and Social Work and Social Policy, School of Allied Health, Human Services and Sport, La Trobe University, Bundoora, VIC, Australia; ^4^Department of Sociology and Social Work, Kwame Nkrumah University of Science and Technology, Kumasi, Ghana; ^5^The School of Arts and Humanities, Edith Cowan University, Joondalup, WA, Australia

**Keywords:** filicide, spirit children, legislation, community intervention, cultural norms, Ghana, cultural filicide

## Abstract

**Introduction:**

Consistent with international promulgation on the criminalization of filicide, Ghana’s Children’s Act 1998 (560) and the Criminal Justice Act criminalizes any form of torture against children. Yet, perpetrators of filicide in Ghana may go unpunished due to the beliefs in cultural norms that justify filicide acts. The cultural narratives of filicide can impede on the application and effectiveness of the laws of filicide.

**Method:**

The study employed a vignette approach to explore the views of 19 adults, who were parents between 69 years of age and 30 years of age, in rural and urban Ghana on the laws of filicide in Ghana and filicide intervention measures. The interviewees were provided with narratives on two different vignettes (developed based on real life cases), followed by semi-structured questions to probe the narratives. The interviews were analyzed following Fraser’s narrative thematic analysis procedure.

**Results:**

The study identifies the association between cultural beliefs and the communities’ understanding of the concept of filicide. Though community members are aware of the criminalization of filicide acts, the majority of them were not informed about the laws against filicide in Ghana. Addressing filicide cases within the community was the most preferred option for the participants, as they believe that some children, termed “spirit children” (SC), deserve to be killed. Resorting to spiritual intervention from concoction men emerged as the normative pathway to obtain community approval for filicide. Police interventions were considered necessary in non-spiritual related filicides. Community members were only prepared to cooperate with the law in filicide cases if the filicide act has no connection with spirituality.

**Conclusion:**

The study adds to understanding of the concept of filicide outside western societies. The importance of intensive community campaigns against filicide acts, and norms that support filicide acts, has relevance for all counties.

## Introduction

Filicide is a form of homicide involving the intentional killing of a child by their parent(s) (cf. Bourget and Labelle, 1992; Butler, 2018; Amon et al., 2019). Perpetrators of filicide face criminal sanctions that are punitive, including life imprisonment, in most societies (Ferguson et al., 2008; Wiest and Duffy, 2013; Klier et al., 2019). Theoretically, punishment of perpetrators of filicide is meant to address the phenomenon by evoking deterrent behaviors. Thus, the application of sanctions against filicide perpetrators will reinforce the normative standards that frown on filicide and violence in general. The legal mechanism of addressing filicide is by far the widely recognized preventive and intervention measure for filicide situations (Amon et al., 2019). However, the application of sanctions against filicide perpetrators may not occur in context where filicide acts may be justified by social norms or cultural beliefs. Unlike most developed countries (for example Australia, United States, United Kingdom, and Canada) filicide cases in Ghana, especially rural areas, are motivated by the beliefs that associate children with severe deformities to the supernatural (Allotey and Reidpath, 2001; Denham et al., 2010; Abdullah et al., 2022). Such children are regarded as spirit children (SC). These children are believed to have links with the supernatural and are sent to cause misfortune to families (Sossou and Yogtiba, 2009; Denham et al., 2010). The justification of severe deformities as SC in rural Ghana may affect the application of legal sanctions against perpetrators. Community members may be reluctant to report filicide to police, when they share in the belief that killing supposed SC is accepted. In contrast to previous studies on filicide in Ghana (Allotey and Reidpath, 2001; Sossou and Yogtiba, 2009; Denham et al., 2010; Abdullah et al., 2022), this study adopted a qualitative narrative approach to explore community’s understanding of laws on filicide in Ghana among rural folks, based on stories of filicide in Ghana. And how filicide cases are addressed in Ghanaian communities.

### Spirit Child Phenomenon in Ghana

In Ghana children who are born with severe deformities, such as (1) having broken limbs (2) complex sexual features (3) born with facial hair or teeth, (4) failing to make eye contact, and (5) crying excessively, are characterized as SC (Sossou and Yogtiba, 2009; Denham et al., 2010). They are considered to have links with the supernatural, and to have deleterious consequences on their family and communities if they live. Community members regard child’s failure to make eye contact as part of the measures these SC’s employ to have their intentions and identity concealed (Allotey and Reidpath, 2001). Mostly mothers are blamed for such misfortunes. It is generally accepted that mothers give birth to such children as a consequences of their involvement in adultery or when they expose their private parts in places where these spirits prevail (Allotey and Reidpath, 2001; Denham et al., 2010). For instance, mothers who have their sexual parts exposed when urinating. Pregnant mothers who expose their bodies are likely to get into contact with these spirits (Abdullah et al., 2022). Traditional healers (*dunsini* in the local Akan language) are known to have the spiritual ability and knowledge to confirm whether a child with severe deformity is an SC (Sossou and Yogtiba, 2009; Denham et al., 2010; Abdullah et al., 2022) and the capacity to provide solution (Adinkrah, 2019). The fate and survival of the supposed SC is dependent on outcome of the consultation with the traditional healer. In addition, the traditional healers provide directives about how the child should be “*sent off*” [killed] (Adinkrah, 2014, 2019). The phrase *sent off* emphasize the notion that these children are regarded as spirits (non-human). A recent study in Malaysia found that perpetrators of filicide used the phrases “*it*” and “*the thing*” to characterize their victim, due to the religious beliefs that supported the killing (Razali et al., 2019, 2020).

It is believed that children who are declared as SC should not have befitting funerals, when they are killed (Denham et al., 2010). They are mostly killed by their biological parents based on directives of the traditional healer. Literature has documented drowning, poisoning, and shooting as the common methods that are used to kill them (Denham et al., 2010; Adinkrah, 2014). It can be evident that this filicide process is motivated by two common factors (1) institutionalization of the powers and responsibilities of the traditional healer, otherwise known as concoction men, and (2) under developed community healthcare system. If these children were born in hospitals, the intervention of the healthcare workers could help sensitize the parents and provide formal intervention. Evidence shows that healthcare infrastructure is on the peripheral stages in rural communities in Ghana (Agbenyo et al., 2017). The effort by Ghana Health Service (GHS) to remedy this situation through the implementation of the Community Based Health Planning and Services (CHIPS) has been hampered by poor roads in most rural areas and limited number of CHIPS facilities (Agbenyo et al., 2017).

Traditional healers have played instrumental role in communities because communities have legitimized their role and status. They provide advice to chiefs and community leaders (including family heads) on matters bordering on divinity. Supernatural powers possessed by the traditional healers are enforced by the collective legitimacy accorded to them, and the fact that some of their predictions do happen. A recent study by Abdullah et al. (2022) revealed that parents who engaged in filicide acts also lost their lives miraculously as predicted by the traditional healer. This, and the legitimacy of their roles, especially advise to chiefs, enforces community’s commitment to their directives. As a result, they often become the first point of contact, and source of intervention when parents suspect their child is an SC. In some cases, the traditional healer uses herbs and traditional medicines to remedy misfortunes that they believe are bound to occur.

For the above reasons, in rural communities, where filicide acts are culturally sanctioned based on the beliefs of SC, community members may not report to law enforcement agencies. Since, the practice of killing SC is normatively sanctioned. Moreover, the community members may have little or no knowledge about the laws that are against filicide acts. Both Parsons (1951) and Gould (1990) identified commitment to legitimized values or norms as a key mechanism that regulates social action. In essence, the willingness to report filicide acts stems from the normative conception of the act as *wrong*, thus against the collective social values, or considered to be culturally *meaningless* (Gould, 2018). It can be argued that even in Ghanaian communities where filicide is less grounded as a crime within local norms there are norms against filicide. Hence community members may likely still report filicide cases to the police and other law enforcement agencies. This is because filicide in these societies may be conceived as deviant and a cruel act that contravenes legitimate norms. Such a scenario depicts what is largely observed in Western countries, where filicide acts have no place in the normative structure of the communities. In Western countries, such as United States, United Kingdom, and Australia filicide is considered a deviant and illegal action, and perpetrators are reported for punishment within the criminal justice system. Evidence from several Western countries have shown that the motivation for filicide varies with the spiritual/cultural interpretations of filicide in Ghana (cf. Flynn et al., 2013; Sidebotham, 2013; Eriksson et al., 2016; Sidebotham and Retzer, 2019). Alcoholism, spouse revenge, parental experience of childhood abuse, mental illness, substance use and unemployment, are common push factors of filicide in the Western world (cf. Flynn et al., 2013; Sidebotham, 2013; Eriksson et al., 2016; Sidebotham and Retzer, 2019). Parents may also kill their children as a result of poverty, whilst fathers use filicide as mechanism to maintain their patriarchal beliefs and norms (Brown et al., 2014). Nonetheless, Sim (2012) report that children with disabilities, in Arizona, are at higher risk of being killed by their parents.

### Legal Sanctions on Filicide in Ghana

Persons convicted of the offense of filicide are liable to be punished through the criminal justice system. Section 11 and 66 of the Criminal Code 1960, (Act 29) and the Criminal Code (Amendment) Act, 2003 (Act 646) of Ghana identify the killing of any person as a form of capital offense, liable to capital punishment. The Act states clear *intention* to kill as constitutive of capital offense. Torture, inhumane treatment and killing of a child is criminalized in the Children’s Act 1998 (560) as well as in the Criminal Code (Amendment) Act, 2003 (Act 646). Specifically, section 13 of the Children’s Act assert that children should not be subjects of any form of torture, punishment or traditional practices that dehumanizes them and affects their mental wellbeing. Even though none of these Acts mentioned filicide categorically, the repugnance of any form of killing, evident in the spirit of the law, shows that acts of filicide may and would be tried in the criminal justice system. The criminalization of filicide concurs with international practices and criminal justice approaches for extreme violence (murder) (cf. Butler, 2018; Amon et al., 2019; Sidebotham and Retzer, 2019).

Because filicide cases are classified as capital offense, offenders are most liable to penalty of murder: death penalty. Accused persons of filicide may be subjected to trial in a court of competent jurisdiction, such as the High Court in Ghana. In Finland, Austria and other developed Western countries perpetrators of filicide are sentenced to life imprisonment (Resnick, 1969; Putkonen et al., 2011; Sidebotham, 2013; Amon et al., 2019). But neonaticide (killing of a baby in the first 24 h) is subject to less severe punishment−often maximum of 2 years imprisonment in most countries. The intention of the severe punishment is to evoke deterrence. However, filicide cases may not be brought to the attention of the court, when community members are unaware of the law, or when they have developed social norms to legitimize their act. Othman et al. (2014) found that filicide cases may not be reported in Ghana due to the cultural beliefs that characterize domestic violence as private matters (Apatinga and Tenkorang, 2021; Tenkorang, 2021). Recent findings from Malaysia, Finland, Austria, Chile and Australia showed that punishment for filicide varies based on gender, with women receiving less severe punishment (Putkonen et al., 2011; Razali et al., 2017, 2019; Amon et al., 2019). Males who committed filicide received higher prison sentences compared to their female cohorts, according to a comparative study in Austria and Finland (Putkonen et al., 2011; Amon et al., 2019). Putkonen et al. (2011) reports that women received relatively less severe punishment for filicide because of the nature of the offense they commit−mostly neonaticide, which is justified by their low level of education, and their status as dependents of other children. Similarly, women who demonstrate extreme remorsefulness sometimes benefits from less severe punishment, such as having 2 years of imprisonment, instead of life imprisonment (Razali et al., 2019).

Effectiveness and application of sanctions against filicide acts is less studied globally. Studies by Charatan (2002); Laporte et al. (2003), and Kauppi et al. (2010) are exception to this. Research on filicide has largely focused on the characteristics and situational circumstances that motivates the act (Brown et al., 2018), with no or little attention on intervention measures for it. Hence, recent studies have advocated for research to focus on unraveling the effectiveness of sanctions as protective and intervention measure for filicide (Amon et al., 2019; Razali et al., 2019) and develop community-based preventive measures to address the risk of filicide (Razali et al., 2019, 2020; Abdullah et al., 2022). Community education, strengthening psychiatric and mental health assessment of people are among the proactive preventive measures documented (Razali et al., 2019; Abdullah et al., 2022). This study builds on the recent findings by Abdullah et al. (2022) to explore community members level of awareness of laws and punishment on filicide in Ghana. It also sought to identify community-based intervention measures, for pre and post filicide acts that are associated with SC and non-SC in Ghana.

## Materials and Methods

### Participants and Procedure

Nineteen adults, aged 30–69, were selected from Ananekrom and Agogo communities in the Ashanti region, Ghana. Because filicide cases are common in rural communities compared to urban communities in Ghana (Allotey and Reidpath, 2001), more than half of the participants (*n* = 12) were selected from the rural community, Ananekrom. The participants were purposively selected through community gatekeepers. The gatekeepers were leaders of the community (assemblymen) who were consulted by the researchers to facilitate the recruitment of participants for the study. It was expected that because the community leaders have lived in the community for long and have had daily social engagements with the community members, they would have reasonable knowledge to lead the researchers to eligible participants. Adults were considered eligible for this study if they have lived in the respective community for at least 6 months. The length of stay in the community may enable them to share their experiences and perspectives on the local norms that regulate filicide practices. The participants were expected to have knowledge about the cultural narratives on filicide in their communities. None of the participants recruited had direct involvement with a filicide case.

The researchers approached the participants with a note containing the objective of the study. The participant information note was read to each participant together with an informed consent form. The rights of the participants to withdraw from the study and decide not to answer some questions were emphasized in the consent form. Participants who agreed to participate in the study were given the prerogative to determine interview date and time. Two of the people who were contacted by the researchers declined to participate, citing personal reasons. Another person declined the invitation as she had a child who is suffering from severe deformities. She cited flash back as the reason for her decision to decline the invitation. The ethical procedure for the study was approved by the Kwame Nkrumah University of Science and Technology.

### Vignette-Based Narrative Interview

The study adopted a vignette-based narrative approach to explore the participants views and experiences on laws on filicide and community-based intervention measures for a supposed SC or non-SC filicide. Vignettes are particularly useful to explore sensitive topics, such as filicide cases, which participants may feel uncomfortable sharing their personal experiences or views on. In research on violence, vignette interviews proceed from the logic of neutralizing negative feelings and avoidance of self-blame. It ensures that both perpetrators of violence and victims are able to describe and share stories about their experiences by way of connecting to an artificial or stimulated argument. Vignettes help researchers to explore issues that may have the potential of creating ethical dilemma (Wilks, 2004). There are ethical concerns when researchers investigate violence cases, including homicide and filicide from the perspective of potential perpetrators or accomplices. The criminalization of filicide acts makes it difficult to explore community members personal/lived experiences of filicide. The vignettes were explored through a narrative-based qualitative approach. A narrative approach emphasizes how meanings are made from stories and narratives of people (Riessman, 2008). Narratives and stories were sought from the participants based on the two vignettes, through a qualitative in-depth interview approach (Silverman, 2013). The approach ensured that participants had the flexibility and openness to share stories including personal experiences on filicide cases in Ghana.

### Instrument

Participants were engaged in in-depth vignette-based narratives interviews at their residence. The interviews were facilitated by two case vignettes (SC and non-SC) and a semi-structured interview questions. The semi-structure questions were used to explore nuances, obtain facts and identify divergence within the stories and narratives shared by the participants. The two vignettes used in this study were developed from two filicide cases that were recently reported in the news in Ghana (see Anim, 2021; GhanaWeb, 2021 for the full case report). The first filicide case (Anim, 2021) involved the killing of a child with sickle cell on the grounds of purported SC, based on directives of a pastor. The second (GhanaWeb, 2021) involved an 11 month old child who was killed by her caregivers after severely maltreating her. The cases highlight two different scenarios of filicide: (1) filicide relating to an SC (SC filicide), and (2) filicide relating to severe physical maltreatment (non-SC filicide). Researchers invited stories on matters leading to the filicide, and what the participants and community will do as a form of intervention. Follow-up questions were asked based on the narratives of each participant to deepen their stories. Examples of some of the follow-up questions included: (1) What can you say about community members’ level of awareness on laws against filicide? (2) In your view, what should parents do if they give birth to a child who has severe deformities that are strange? (3) What factors do you think contribute to parent’s decision to decide to kill a child? (4) What form of sanctions are given to those who engage in the filicide act?

Interviews were conducted at the residence of the participants using the Twi language (the common local language in Ghana). The interviews were facilitated by the researchers, who are trained social workers, and have expertise in qualitative interviewing. The interview spanned 90 min on average, and were audio recorded. Interviews were conducted between October to December 2021. Each interviewee was given an incentive of 30.00 cedis (∼$4.00 at the time of the interview) to compensate for their voluntary participation.

### Analysis Approach

Narratives from the participants were transcribed verbatim. The transcripts were read three times by three members of the research team to ensure accuracy and to familiarize themselves with the data. The data was analyzed following Fraser (2004) suggested thematic analysis procedure for narrative interviews. First, each narrative was organized to constitute individual stories and sub-stories. Sub-stories were created following segmentations within the narratives. Phrases were used as labels for each segmented story. Phrases such as “not a matter of police” “spiritual man determines it” “they are not human” were common. The segmented stories and labels (phrases) were considered critically at three domains: personal, interpersonal and cultural domains (Fraser, 2004). At the personal level stories were interpreted according to the personal beliefs of the participants. Stories were analyzed based on the connection between participants and others in their social network (interpersonal level). Stories were also considered in line with the cultural beliefs and practices in the community that supported or opposed filicide. The cultural domain was crucial in analyzing and making sense out of the stories due to the cultural connotations and implications of the narratives. These domains provided context to make sense out of the data and guided the researchers to draw meaningful implications from the stories. Themes were created based on commonalities, coherence and chronology in the segmented stories. In creating themes, key attention was paid to nuances in the stories, mostly evident in divergent opinions. The final themes and associated segmented stories were keenly discussed among the researchers. Minor changes were made in the themes following the review by the entire research team. The entire analysis process was facilitated by Taguette software.

## Findings

### Demographic Summary of the Participants

The participants included 12 females and 7 males within the age bracket of 30 to 69. The average age among the 19 participants was 48. Overall, the participants spent an average of 36 years of living in the same community. The extent of neighborhood stability has implications on the participants’ level of experience and depth of knowledge about the cultural practices that support filicide acts in the community. Only four of the participants had a Bachelor’s degree, with two of them identified as retired health practitioners. The remaining participants were peasant farmers (*n* = 10) and traders involved in sole proprietorship business (*n* = 5), who had Senior High School as their highest educational qualification.

### Themes

Key themes that emerged from the stories and narratives of the participants have been presented in this section. The themes are presented in line with the focus of the study and discussed based on the nuances in the narratives.

#### Knowledge on Laws of Filicide

##### Killing of Children Is Prohibited

All the participants agreed that killing your own child as well as any other child is prohibited and criminalized in Ghana. However, they had no knowledge about the specific regulations or laws that prohibit filicide in Ghana. Reacting to the vignette on non-SC filicide, a participant had this to say:

“*Most people are aware that the laws of Ghana frown on killing people irrespective of their age. You will be prosecuted if found doing that*” (P1, rural community).

An important caveat was that participants who reacted to the vignette on SC argued that engaging in filicide based on SC will not be considered as filicide because it is an act of *send-off*, not actual killing of a human being.

“*I am aware that when you kill someone you will be jailed but in the case of these children, they are sent off to the spiritual world. It is just like seeing a visitor off. The spirits have their own world they live in. We cannot accept and live with them*” (P4, rural community).

The severe consequences that could befall on the families are used to justify the killing of the child who is born with severe deformities. A participant in the urban sample shared below:

“…*there are some consequences that might follow if you decide to keep such children and raise them. It is therefore important that when a child of such nature is born, the appropriate traditional rites are performed so that they leave us in peace. You cannot term this as killing. They are not humans, they are from the spiritual world and should be made to go back*” (P11, urban community).

It appears that parents use the term spiritual send-off to protect against the risk of facing the law for engaging in filicide. Another participant in the urban community shared this:

“*I also believe that because of the knowledge on the laws of killing a child, they would not want to term their actions as killing but rather a spiritual send-off. When it is considered a spiritual send-off no one will report to the police because everyone accept that it is the only way to send such children back to where they came from*” (P19, Urban community).

##### Laws Cannot Handle Spiritual Issues

The process of killing children who are confirmed as SC is described as a spiritual process, which is sometimes difficult to witness. According to participants in this study the spiritual and miraculous nature of the process makes it hard to establish evidence against the filicide offenders. A participant in the rural community gave brief account of the process involved:

“*When a spirit child is being ‘sent off,’ spiritual men are engaged. They perform a lot of spiritual activities that cannot be described or seen with the human eye. This makes it difficult to establish substantive evidence to help prosecute people like that*” (P15, rural community).

Her opinion was echoed by another participant who suggested that even if someone volunteers to make a report, it will be difficult to identify the culprit for them to be prosecuted within the criminal justice system.

*“Issues of spiritual nature are not addressed by the law. Even if there is a volunteer who is willing to point out people who carried out such exercise, it will be very hard to prosecute them in court. Although the parents lead the process by making a report about the child’s deformities and conditions, the actual process involve some family members, close friends, led by the concoction men of the community”* (P16, urban community).

This suggest that the act of filicide, based on SC, may be institutionalized in some Ghanaian communities as it is carried out by parents, kith and concoction men.

#### Addressing Spirit Child Phenomenon

##### Assistance From Spiritual Leader

Reacting to the vignette on SC filicide the research participants shared stories about the role of spiritual leaders. They argued that spiritual leaders (including the concoction men and religious leaders) are the first point of contact by families when they give birth to children with severe deformities and ailment that are not common to the community members. Intervention from the concoction men or spiritual leaders mostly lead to the killing of the children. However, in some cases, they provide herbal medications which help to cure the disease. One participant shared this:

“*Not all children who are considered spirit children develop such traits at their early stages of birth. Some are identified a year after birth and this makes the parents associate the reaction with dark forces that are working against them. To solve such mess, parents usually consult spiritualist or pastors to find remedy for their children. So, not in all cases that the child will be killed. The traditional healer can help remedy these spiritual happenings through traditional herbs. Even though in most cases the children are killed based on the directive of the traditional healer, sometimes they survive*” (P8, rural community).

For the children whose continues existence are interpreted to have consequences on the family, the spiritual leaders recommend for them to be killed. A participant shared a story drawing on her personal experience of a parent who experienced severe hardship as a result of giving birth to a child with seven fingers:

“*In the Akan traditional setting, there are some births which are considered taboo. In relation to this, when a child is born with severe deformity or strange features, it is believed that they are from the spiritual realm. This means that the assistance of an individual who has knowledge in spiritism must be consulted to help create a safe passage for the child to return. Failure to conform to this, the parents are made to believe that things would not augur well with them either financial or in their marital life. I know a woman who experienced severe hardship ‘sika 3ntina ne nsam’ [literally meaning money doesn’t stay in her hand] after giving birth to a child who had seven fingers instead of five. It is believed that the child is a spirit child and ought to be sent off at an early stage. However, because she did not agree, she has been made to suffer such atrocities by the spiritual parents of the child*” (P15).

The idiom “*sika 3ntina ne nsam*” is used to describe financial hardship associated with spiritual happenings.

Narratives from the participants suggest that consultations with the spiritual leader is necessary as most of the ailments associated with SC happen due to spiritual curse to the parents. The following stories by two participants highlight how this happen and diversity within.

“*I know of a woman who gave birth to a spirit child because she urinated in a river when she went to the farm. People in this town say she was cursed by the river goddess for disrespecting her. Rumor has it that, the woman was pregnant at the time. I personally believe that, during pregnancies, the child in the womb is vulnerable to spiritual attacks and so the least provocation would lead to the destruction of the child. Once the child is born, the curse must be revoked so the child can return peacefully or survive as a normal child*” (P17, rural community).

“*In the early 80s, I was a resident in Sekondi. A woman had a baby who was too heavy for the size of a normal child. The community believed that the child was cursed by the Gods of the sea. This made the family of the woman seek assistance from community spiritualist to revoke the curse. The result of this act was that, the woman had to lead the spiritualist to the sea with mashed plantain and other materials. At the shores of the sea, the woman was made to turn her back to the child and gunshots were fired. After this, the woman was made to leave the site without looking at the child. It is believed that once she looks back, she would give birth to the spirit child again*” (P11, urban community).

The final narrative suggests that in some cases the act of killing the child is performed by the spiritualist or traditional healer. Which reiterate the notion that filicide practices are legitimized in some communities and supported by traditional cultural beliefs. This was confirmed by narratives from another parent:

“*I witnessed an extreme case in Walewale in the Northern region of Ghana. This was during my early stages of professional practice in the town. Once a baby is delivered with such deformities, the spiritual head of the community is informed first. They then storm the health facility to request the baby from the parents and take the child to the shrine for spiritual incantations. In most cases, the parents may not see the child again. In cases where the spiritual head is not informed, the family of the child would personally go to the shrine to seek clarification on issues before they accept the child in the house*” (P12, Urban community).

The narratives highlight ways community beliefs on SC enforce filicide practices.

##### Child Isolation Due to Stigma

In context of the community beliefs and practices that strengthen filicide acts, some parents isolate their children from the public to ensure that they are not adjudged as SC, and forced to kill them. Children with severe deformities are also protected from stigma when their parents isolate them from the community members or the general public. Narratives from two participants highlight this safeguarding mechanism:

“*Due to the stigma attached to having spirit children, parents who are bold enough and educated on the circumstance surrounding the condition of their child mostly keep them away from society. They either lock them in rooms where no one has access to, or they keep them indoors when there are visitors in the room*” (P4 rural community).

“*There is a woman who live next to our junction here. She has a child who is autistic and experiences severe meltdown as well as panic attacks. If the child goes out people will begin to associate her to SC. They will start to point hands at the family saying that they are cursed. So, she has decided that no one will have contact with the child. Which has helped. The child is 12 years now and he is doing well. He would have been forced to see the spiritual man, if she had allowed the child to go out*” (P2, urban community).

Another participant shared that as part of the safeguarding measures, parents who give birth to children with deformities send them to special schools for them to be cared for. However, she reiterated that only the rich can afford the cost involved.

“*The rich people mostly send them to special schools for them to be raised there. One of our directors had a child like that. Even with his status as a well-paid government worker, he could not live with the child. He has sent the child to a residential home in Accra, which is a special facility for such children. He hardly visits the child. He however sends feeding money and other things for the upkeep of the child. The stigma that comes with the child’s state has prevented him from integrating the child with the family. Some members of the local church even believe a demon possessed the child in one of their prayer meetings. This even influenced his decision to send the child away*” (P18, urban community).

##### Police Intervention in Non-spirit Children Filicide

According to the participants filicide incidents that are not related to SC will be reported by community members when they witness. Stories about the non-SC vignette evidenced the fact that community members frown on parents’ intention to kill their own children for reasons other than SC related. The narratives below summarize assertion from the participants in this study:

“*When a parent decides to kill their own child for reasons unknown to the community, she is likely to be reported to the police. I have witnessed an incident where a teenage mother was arrested for throwing her child into the public latrine. Later it came to the limelight that, the teenage mother despised the baby and hence wanted to give her away*” (P7, urban community).

Non-SC related filicide are characterized as parents’ sole intentions to kill their own children.

“*Not all incidence of filicide are spiritually related. Some are based on intentions of parents. Such behavior would not be entertained by the society. People around will definitely report this to the police. It is even a bad omen for a parent to have ill thoughts for his/her child*” (P3, rural community).

## Discussion and Implications

This study sought to unravel community members level of awareness of legislation on filicide in Ghana, and the intervention efforts they undertake before and after the filicide incident. Findings from vignette-informed narrative interviews adds to existing body of knowledge on filicide in Ghana. It also draws attention to the unique differences between intervention for SC and non-SC related filicide.

Evidence from this study show that parents in both rural and urban communities in Ghana accept that killing of biological child(ren) is against the laws of Ghana. However, they could not specify the regulations and sanctions that will be meted on perpetrators of filicide. Both the Criminal Code (Amendment) Act, 2003 (Act 646) and the Children’s Act 1998 (560) of Ghana provide generic statements that frown against the killing of anybody, including children. But they do not mention filicide acts. Section 11 and 66 of the Criminal Code (Amendment) Act, 2003 (Act 646) characterizes any act involving the killing of a fellow human as a capital offense. Which is subjected to capital punishment. Considering the increasing cases of filicide in Ghana (Allotey and Reidpath, 2001; Sossou and Yogtiba, 2009; Denham et al., 2010; Anim, 2021; GhanaWeb, 2021), it is important to raise awareness on the legal consequences of filicide and the legal instruments should be amended to include specific regulations on filicide. Such amendments should particularly address the issue of SC related filicide. Participants in this study confirmed that children who are killed on the basis of being accused as SC, should not be considered as “killed.” Instead, such children are *sent-off* to their spiritual source. The term spiritual *sent-off* has appeared in most studies on filicide in Ghana (Sossou and Yogtiba, 2009; Denham et al., 2010; Adinkrah, 2014, 2019; Abdullah et al., 2022). It appears as the accepted *quia* [vocabulary] to normalize SC-related filicide in Ghana. It was not surprising that participants in this study argued that their communities do not perceive SC-related filicide as actual filicide. Instead, they perceived it as a form of spiritual send-off, because such children belong to the spiritual realm. In addition to advocating for a proposed legislation to ban all forms of SC-related filicide in Ghana, it is important to also implement normative change programs in communities to change the deep-rooted norms on SC filicide.

A recent report by Anim (2021) suggest that some communities in the northern part of Ghana have developed local by-laws against SC-related filicide. These community-based initiatives are important to address the phenomenon of SC and related filicide in Ghana. The initiate has the promise of strengthening legal sanctions against filicide whilst changing the normative justification of such acts. Narratives from the participants in this study has shown that laws on filicide, as a result of SC, may not work in the current judicial system of Ghana due to the challenges in dealing with spiritual matters within the court of law. However, because the characterization of SC filicide as spiritual *send-off* is socially defined within the normative structure of the communities (Allotey and Reidpath, 2001; Sossou and Yogtiba, 2009; Abdullah et al., 2022), community-based initiatives are required to address the behavior. It is suggested that the community-based programs should first re-characterize SC-related filicide as non-spiritual filicide and actual killing of a child. When this is achieved, the normative order on SC and filicide would be reshaped. Changing the meaning of killing of SC can facilitate community members accepting SC-related filicide as a non-normative conduct which deserves to be punished. The findings of this study have shown that community members are willing and ready to report non-SC filicide to the police. Therefore, when SC is accepted as a fallacy, and deceit, the phenomenon of filicide in Ghana will reduce significantly.

The study’s findings indicate that the characterization of SC is related to spiritual beliefs or curse. Parents who give birth to supposed SC are regarded as being cursed by the god’s. Evidence from several studies in Ghana have shown that curse is a major attribute of SC related issues (Allotey and Reidpath, 2001; Sossou and Yogtiba, 2009; Denham et al., 2010; Adinkrah, 2019). Like Sossou and Yogtiba (2009) and Denham et al. (2010) it emerged that most of the children who are characterized as SC suffer from diseases and impairments that affect their normal development. Autism, down syndrome were among the common diseases associated with SC. It appears that these children are killed because of community member’s lack of understanding of such diseases. SC filicide may be reduced and curtailed when community public health education is intensified in rural communities. Public health education should focus on teaching (1) symptoms of these diseases (2) treatment mechanisms, and (3) common behaviors exhibited by children who suffer from them. CHIPS should be established across the country to intensify the public health education (Agbenyo et al., 2017). Home births without the presence of a professional health worker should be discouraged. Our data has shown that increase in hospital delivery could help save the lives of most children who are born with autism and other diseases that affect their development. Antenatal care for pregnant women, at regular intervals, should be encouraged in rural communities to prevent congenital abnormality leading to the birth of severely deformed children.

The study evidenced isolation and special schools as necessary protective intervention measures that are used by parents to prevent their children from being killed or stigmatized. The use of special schools for children with autism and other developmental diseases is widespread (Scott et al., 2002; Reed et al., 2012). It can be a meaningful short-term intervention measure, before efforts are made to create an inclusive system that ensures the integration and acceptance of children with autism and other developmental diseases.

### Limitation

Several limitations apply to the study. First, the use of specific case vignettes may direct the interviews toward specific focal issues, and limit the participants. Even though, follow-up techniques were effectively employed, the depth of insight and diversity of the interview could vary when different vignettes are used or when non-vignette open semi-structured interview approach is adopted. Nonetheless, the vignette proved very useful to examine a delicate and sensitive topic of this nature. A sample of 19 is very large qualitatively, but not reasonable to draw statistical inferences from the data. The selection of participants from communities in the Ashanti region may have biased the findings, in terms of the cultural underpinnings of filicide. Studies from other regions, particularly northern part of Ghana, are needed to explore nuances in the intervention measures for filicide cases. The inclusion of mostly uneducated and less educated participants may suggest that the findings represent the views of all lay people in rural areas. Research is needed to unpack these cultural narratives from the perspectives of educated parents living in urban areas. Further studies could explore the necessary sanctions that can deter SC filicide in Ghanaian communities.

## Conclusion

Filicide is an internationally recognized cruelty against children, and a major contributor to child homicide. The phenomenon of filicide, particularly SC filicide, has gained traction in recent media discussions in Ghana. This sought to explore community members level of awareness on laws against filicide acts in Ghana. It also explored intervention measures that are undertaken by community members pre and post filicide acts. Our findings reveal two main categorization of filicide; SC and non-SC filicide. The former is not considered as filicide because communities have legitimized the belief that children with severe deformities, who are labeled as SC, are not actual human beings. Hence, they are only *sent-off* to their spiritual source when killed. This categorization has institutionalized a norm of non-response and actions from community members when children are killed on grounds of SC. Community-based initiatives backed by local byelaws and legal sanctions are recommended as important steps to change the normalization of SC filicide. The study findings contribute significantly to our understanding of the cultural underpinning of SC and filicide in Ghana and Africa at large. It highlight measures that are required to address the nuanced categories of filicide in Ghanaian communities. Findings from the study provided narratives to inform direction for addressing filicide in a society where traditional cultural and spiritual beliefs in local communities are associated with attitudes to filicide and factors which are used to justify the act in specific circumstances. The findings highlight the importance of formulation of targeted filicide legislations particularly to aim at addressing the spirituality aspects of filicide in those societies where this occurs.

## Data Availability Statement

The datasets presented in this article are not readily available because data identifiable therefore cannot be shared. Requests to access the datasets should be directed to MF, m.frederico@latrobe.edu.au.

## Ethics Statement

The studies involving human participants were reviewed and approved by the ethical procedure for the study was approved by the Kwame Nkrumah University of Science and Technology Ghana. The patients/participants provided their written informed consent to participate in this study.

## Author Contributions

AA conceptualized and designed the study, collected data, drafted the initial manuscript, and reviewed the manuscript. MF conceptualized and designed the study, coordinated and supervised data collection, drafted section of the discussion, and critically reviewed the manuscript. FM conceptualized and designed the study, carried out the data analysis, drafted analysis and methods section of the manuscript, and reviewed the manuscript. HB conceptualized and designed the study, coded the interview, drafted the initial manuscript, and reviewed the manuscript. YW conceptualized and designed the study, data analysis, and reviewed the manuscript. JA conceptualized and designed the study, drafted section of the discussion, and critically reviewed the manuscript. All authors approved the final manuscript as submitted and agreed to be accountable for all aspects of the work.

## Conflict of Interest

The authors declare that the research was conducted in the absence of any commercial or financial relationships that could be construed as a potential conflict of interest.

## Publisher’s Note

All claims expressed in this article are solely those of the authors and do not necessarily represent those of their affiliated organizations, or those of the publisher, the editors and the reviewers. Any product that may be evaluated in this article, or claim that may be made by its manufacturer, is not guaranteed or endorsed by the publisher.
